# Adhesion force microscopy is sensitive to the charge distribution at the surface of single collagen fibrils†

**DOI:** 10.1039/d2na00514j

**Published:** 2022-10-18

**Authors:** Vinayak Mull, Laurent Kreplak

**Affiliations:** Department of Physics and Atmospheric Science, Dalhousie University Halifax Nova Scotia Canada kreplak@dal.ca +1 902 494 8435

## Abstract

Collagen fibrils are a key component of the extracellular matrix of mammalian tissues where they serve as structural elements and as a ligand for receptor-mediated signaling. As collagen molecules assemble into fibrils, *in vitro* or *in vivo*, they acquire a modulation of their molecular and electron densities called the D-band, with a 67 nm spacing, that can be visualized by cryo-electron microscopy. The D-band is composed of a gap region missing one-fifth of the molecules in the cross-section compared to the overlap region. This leads to the gap region having a positive potential and the overlap region a negative potential with respect to an n-doped silicon probe as observed by Kelvin Probe Force Microscopy. In this study, we use the adhesion force between an n-doped silicon probe and a collagen substrate to demonstrate the sensitivity of adhesion force towards charge distribution on the surface of collagen fibrils. We also map the charge distribution at the surface of single *in vivo* and *in vitro* assembled collagen fibrils and characterize the three-dimensional location and strength of three sub D-band regions that have been observed previously by cryo-electron microscopy. Our approach provides an adhesion fingerprint unique to each fibril type we analyzed and points to local charge variations at the sub D-band level even along a single fibril. It opens the road for a detailed analysis of collagen fibrils surface modifications due to ligand binding or the accumulation of advanced glycation end products at sub D-band resolution on a fibril by fibril basis.

## Abstract 2

Charges at the surface of collagen fibrils modulate adhesion force measured by the AFM even in the absence of an applied voltage.

## Graphical abstract

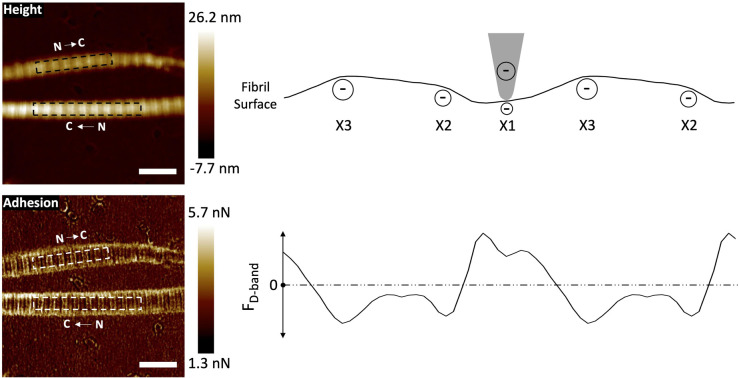

## 1 Introduction

**Fig. 1:**
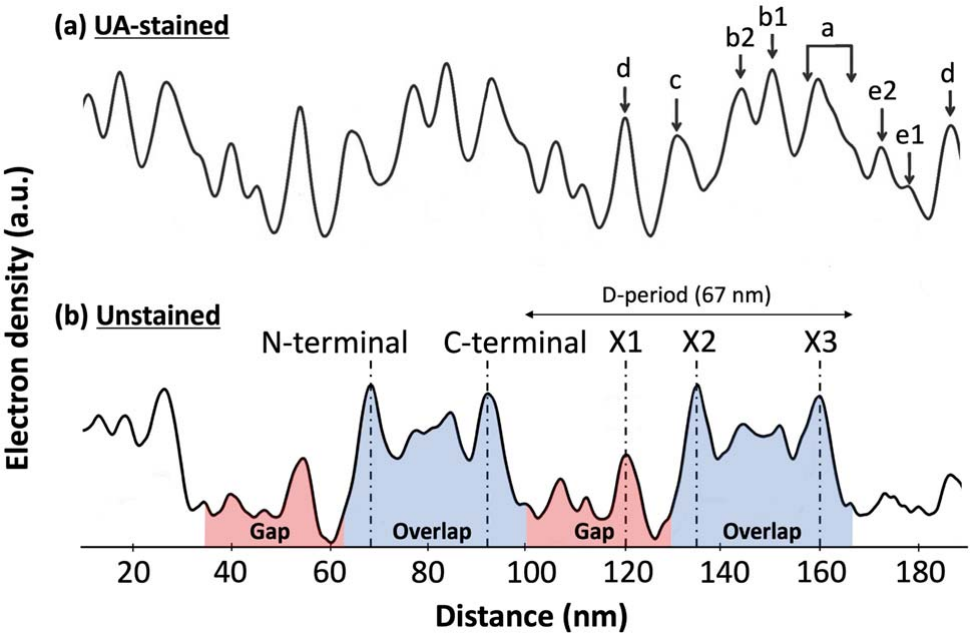
Electron density fluctuation along a rat-tail tendon collagen fibril observed by cryo-electron microscopy, adapted from Quan and Sone.^7^ (a) Uranyl Acetate (UA)-staining profile along the fibril reveals regions rich in negatively charged amino-acids within the fibril. (b) In the unstained profile, three main electron dense peaks are visible within the 67 nm D-band repeat, the X1 in the gap region and the X2 and X3 in the overlap region coinciding with the N- and C-terminal domains of the collagen molecule, respectively.

Collagen is the most abundant protein in the mammalian body. It is a major component of the Extracellular matrix (ECM) where it acts as a fibrous scaffold, providing structure and integrity to connective tissues like tendons and bones.^1^ Collagen fibrils are formed by the assembly of collagen triple-helical molecules into a staggered para crystalline array.^2^ Molecular staggering leads to the formation of a characteristic overlap region, where all the molecules are present in the cross-section, and a gap region, where 20% of molecules are missing. A consecutive gap and overlap pattern is called a D-period or D-band, which is approximately 67 nm in length. Within a D-band, molecular staggering originates from a repeating sequence of positive and negatively charged residues along the collagen molecules providing collagen fibrils with a unique charge profile that was first demonstrated by negative staining transmission electron microscopy.^2,3^ This charge pattern is essential for the proper assembly of collagen fibrils and explains the sensitivity of *in vitro* assembled fibrils' morphology to changes in pH and ionic strength.^4,5^ Experimentally, there are different ways of characterizing the electron density within a fibril without staining, small angle X-ray scattering that led to the only atomic model of the collagen fibril's interior,^6^ and cryo-electron microscopy that revealed three main electron-dense regions within a single D-band repeat of rat-tail tendon collagen fibrils (Fig. 1).^7^ It is worth mentioning that this is a bulk electron density variation and that molecular remodeling is expected to alter the distribution at the surface of the fibril thus revealing cryptic binding sites for protein ligands.^8^Fig. 1 also shows the Uranyl Acetate-staining profile along the same profile. Uranyl acetate binds to the negatively charged amino acids, Aspartate, and Glutamate in the fibril, so this stain profile represents the variation of localized negative charges, and peaks indicate regions with a greater number of negative charges than the immediate surroundings. Whether or not these bands have a net charge is still an ongoing question. Considering the constellation of binding sites predicted by atomic scale models of the fibril surface,^9,10^ direct measurement of the charge distribution at the surface of the fibril is attracting renewed attention.^11^

Kelvin Probe Force Microscopy (KPFM) is one of the available techniques to image surface charges at the nanoscale.^12^ In the AM-KPFM mode, the AFM first scans the topography of the sample with a simple scan under amplitude feedback. Then, the AFM probe is lifted to a given height above the surface to perform a second scan at the natural resonant frequency of the cantilever, following the trajectory acquired during the topography scan, without feedback. During this second scan, a DC voltage is adjusted throughout to zero the oscillations induced because of the electrostatic force between the tip and the sample caused by the surface potential and applied alternating voltage. This leads to the measurement of the Contact Potential Difference (CPD)^13,14^ and in turn, the charge distribution at the surface. KPFM images of rat-tail tendon fibrils reveal a surface potential modulation corresponding to the D-band where the overlap region is more negatively charged, *i.e.* lower potential, than the gap region.^15^ The images, however, lack sub D-band features seen by cryo-EM (Fig. 1), which is likely because the measurement is not taken in contact with the sample and thus, the electrostatic force is not isolated to only the tip apex, as it also interacts with the tip pyramidal shape and the cantilever shaft. Since the CPD measurement is an average of all of these electrostatic contributions, this results in a low resolution of about 50 nm in current AM-KPFM measurements.^14^

In principle, for localized charges implanted at the surface of a dielectric material, it should be possible to map the charge distribution by simply measuring the adhesion force between the pyramidal tip of an atomic force microscopy (AFM) probe and the sample. One of the components that this adhesion force is dependent upon is the electrostatic force between the tip and the sample. This electrostatic interaction varies according to the charge distribution on the surface. In the case of a conductive tip and a conductive or naturally charged surface like mica, a voltage bias can be used to increase the adhesion force by several orders of magnitude through the attractive electrostatic interaction of opposite charges.^16^ This effect has previously been observed on a hydrophilic surface at a low relative humidity of below 40% RH even in the presence of water absorption and the associated screening of the electric potential.^16^ In the absence of a voltage bias and at low relative humidity, it is still expected that the adhesion force between a charged silicon tip and a charged surface should depend on the spatial distribution of charges. Considering a negatively charged tip, a flat surface with bound charges, and ignoring the effect of surface roughness,^17^ localized negative charges should yield a decrease in the total adhesion force whereas localized positive charges should yield an increase in the total adhesion force.

**Fig. 2:**
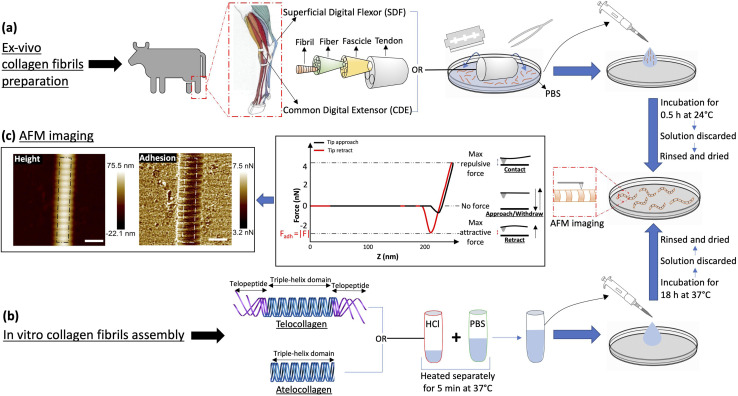
Experimental protocol. (a) Collagen fibrils are extracted by scraping either Superficial Digital Flexor (SDF) or Common Digital Extensor (CDE) tendons in PBS. The solution containing fibrils is transferred to a Petri dish for incubation at 24 °C for 30 min, which thereafter is discarded and the dish is rinsed with ultra pure water and dried with N_2_ gas. (b) Atelo- or telocollagen molecules stored in 0.01 N HCl and PBS solution are heated separately at 37 °C for 5 min, then mixed and incubated in a Petri dish at 37 °C for 18 h. Similar to (a), the solution is thereafter discarded and the dish is rinsed and dried. (c) Collagen fibrils adhered at the bottom of the dish are imaged by atomic force microscopy in peak-force mode. A typical force–distance curve highlights how the attractive adhesion force between the tip and the sample is measured at each pixel of the image along with the height. Several pixel wide profiles (white dotted lines) are extracted from these images for analysis. (Scale bar = 200 nm).

Leveraging this fact and the periodic nature of the collagen fibrils' surface charge distribution, we acquire adhesion force maps of collagen fibrils in an ambient condition at a pixel resolution of 4 nm for four different types of collagen I fibrils, assembled *in vitro* from collagen molecules with and without their charged telopeptides (telo- and atelo-collagen, respectively), and extracted from two bovine leg tendons with functionally distinct nanostructures (Fig. 2).^18^ We show that adhesion force fingerprints extracted from the images reveal three electron density sub-bands similar to the ones observed by cryo-electron microscopy (Fig. 1) with significant variations in strength and position independent of height variations. Considering the main forces involved and upon observing consistency between our results and others', we hypothesize that the n-type silicon tip used in our experiment acts like a negative charge. Our results support the idea that the charge distribution at the surface of the fibril is significantly different from the interior of the fibril and depends on the presence of the telopeptides as well as the anatomical location of the fibril.

## 2 Materials and methods

### 2.1 *Ex vivo* collagen fibrils preparation

For *in vivo* collagen fibril isolation, a matched pair of Common Digital Extensor (CDE) and Superficial Digital Flexor (SDF) tendons were procured from the leg of a steer at a local abattoir. These dissected tendons, stored at −80 °C, were cut and used as needed, as follows (Fig. 2a). A tendon piece was quickly transferred on a Petri dish in 500 μL of Phosphate Buffered Saline (PBS), and then scrapped with the help of a blade and tweezers to isolate collagen fibrils from the tendon into the solution. The tendon was discarded and the solution was transferred to a glass-bottom Petri dish where it was incubated for 30 minutes at room temperature to let the fibrils adhere to the bottom of the dish. The solution was discarded and the dish was rinsed four times with 5 ml of ultrapure water and finally dried with nitrogen gas.

### 2.2 Collagen fibrils self-assembly

*In vitro* collagen fibril assembly was carried out using 3 mg ml^−1^ Type-I atelo- and telocollagen solutions in 0.01 N HCl, extracted from bovine hides (Advanced Biomatrix, USA) (Fig. 2b). First, the collagen solution in acid and PBS were warmed separately in a water bath at 37 °C for 5 minutes. The two solutions were mixed together to final concentrations of 200 μg ml^−1^ for telocollagen and 300 μg ml^−1^ for atelocollagen, transferred to a glass-bottom Petri dish, and incubated at 37 °C for 18 hours. The solution was then gently pipetted out and the dishes were gently rinsed with 5 ml of ultrapure water four times, followed by drying with nitrogen gas.

### 2.3 Atomic force microscopy

Collagen fibril samples were imaged on a Bioscope catalyst (Bruker, USA) atomic force microscope mounted on an IX71 inverted optical microscope (Olympus, USA) and operating in Peak Force Quantitative Nanomechanical Mapping mode (Peak Force QNM). The cantilevers used were made of silicon nitride and had an n-doped Silicon pyramidal tip with a nominal radius of 2 nm and a half angle of 18° (SCANASYST fluid+, Bruker USA). Note that in this architecture, the pyramidal tip behaves as an insulated conductor that is not necessarily grounded. The spring constant was calibrated for each cantilever before imaging using the thermal noise method and ranged from 1 to 1.5 N m^−1^. 1 μm images were acquired with 512 pixels per line at a scan rate of 0.5 Hz, peak force setpoint of 5 nN, cantilever oscillating frequency of 1 kHz and corresponding vertical tip velocity of 0.6 mm s^−1^. Two channels were recorded for analysis, the height and the adhesion force between the tip and the substrate (Fig. 2c). For all the images, the humidity in the room was between 15 and 20% RH.

### 2.4 Data analysis

**Fig. 3:**
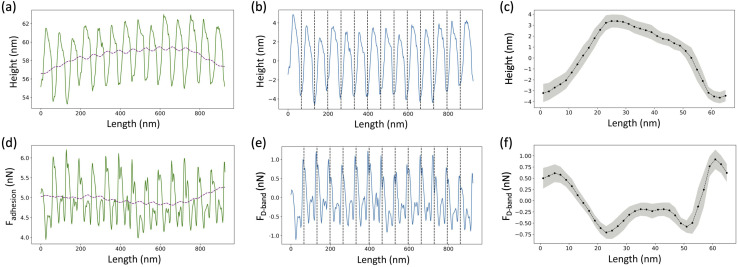
D-band averaging. (a) A profile extracted along the length of the fibril (solid line) is used to calculate an adjacent average with a window of size equivalent to 3 D-band repeats of 67 nm each (dotted line). (b) The filtered profile (solid line), obtained by subtracting the adjacent average from the extracted profile, is Fourier transformed to calculate the value of D-band repeat and then divided into individual D-band periods (dashed lines). (c) All individual periods are averaged together to obtain a height fingerprint of the D-band. (d) A profile synchronized with the one taken from the height image is extracted from the adhesion image (solid line), which measures the total adhesion force, *F*_adhesion_ experienced by the tip. Following the same process, an adjacent average is calculated (dotted line) and subtracted from the extracted profile. (e) The resultant filtered profile (solid line) corresponding to the adhesion force due to the D-band only, *F*_D-band_, is divided into individual D-periods (dotted lines) using the value of the D-band repeat obtained from the paired height profile. (f) The D-periods are averaged together to create the adhesion fingerprint of the D-band. The grey shading in (c) and (f) represents the standard deviation of the D-band periods with respect to the fingerprint.

For the height images, a 1st order plane fit followed by a 1st order line by line polynomial fit, with the fibril masked, was applied using NanoScope Analysis (Bruker, USA) in order to flatten the image. A several pixels wide profile, ranging from 28 pixels to 107 pixels depending upon the fibril diameter, along the length of the fibril was extracted using SPIP (Image Metrology, Denmark) (Fig. 3a, solid line). The following analysis was done using a Python Script. An adjacent average of the height profile with a window size of 103 pixels, which is equivalent to 3 D-band repeats, 67 nm each, was computed (Fig. 3a, dashed line) and then subtracted from the profile to remove previously reported long-range fluctuations in height not associated with the D-band repeat.^19^ The obtained filtered profile (Fig. 3b) was Fourier transformed to obtain the value of the D-band repeat and sliced into individual D-band periods (Fig. 3b, dashed lines). Finally, all the individual height profiles were averaged together to obtain the height fingerprint of the D-band (Fig. 3c). A similar process was followed for analyzing adhesion images except that the value of the D-band repeat obtained from the paired height profile was used for slicing the D-band periods of the adhesion profile (Fig. 3e–g). This was possible because the profile extraction from the height and adhesion images was synchronized using SPIP. The final adhesion fingerprint obtained by removing the adjacent average from the adhesion profile displays the change in adhesion force, *F*_D-band_ attributed to the D-band only.

## 3 Results and discussion

### 3.1 The adhesion force along a collagen fibril is insensitive to nanoscale topography

**Fig. 4:**
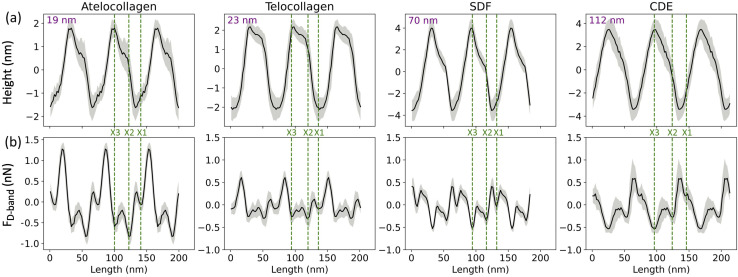
(a) Height and (b) Adhesion fingerprints for four different collagen fibrils obtained from atelocollagen, telocollagen, SDF and CDE samples. Each fingerprint is repeated over three D-band periods for clarity and includes the average of all the D-periods along the fibril (solid line) as well as the standard deviation between individual D-periods (grey shade). In addition, the average height of the fibril is indicated as well as the proposed positions of the three main electron density bands, X1, X2 and X3 (dotted lines) observed by cryo-electron microscopy (Fig. 1).

In this study, collagen fibrils extracted from two tendon types, SDF and CDE, and fibrils self-assembled *in vitro* from atelo- and telocollagen molecules (Fig. 2), were imaged by adhesion force microscopy at a relative humidity between 15 and 20%. The fibrils had a wide range of height, between 11 nm and 149 nm, and exhibited the expected D-band repeat with a period of 66.5 nm (SD = 3.4 nm) (*n* = 30). Height fingerprints (Fig. 4a) were obtained by a process of filtering and averaging of profiles extracted from height images (Fig. 3).

**Fig. 5:**
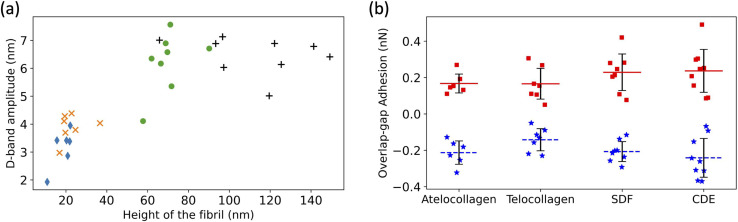
(a) Increase in D-band amplitude with height of the fibril. atelocollagen (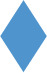), telocollagen (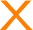), SDF (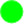), and CDE (+). (b) Overlap adhesion (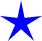) and Gap adhesion (■).

The D-band amplitude, measured using these height fingerprints, showed a positive correlation with the height of the fibril-flatter, *in vitro* fibrils have smaller D-band amplitude as compared to the taller, *ex vivo* fibrils (Fig. 5a).

An almost identical trend of D-band amplitude with fibril height has already been reported for collagen fibrils extracted from bovine corneal and scleral tissues.^20^ As the height of the fibril and the amplitude of the D-band decrease, the overlap region of the height profile displays clearer ridges, as seen by comparing the CDE and atelocollagen examples (Fig. 4a). This increase in visibility of sub D-band features with a decrease in height is in agreement with a previous imaging study on 3 nm tall self-assembled collagen tapes, which revealed up to twelve sub D-band features within a single repeat.^21^ The adhesion measurements, however, did not reflect a similar trend with height. There was no significant difference between the mean adhesion of each fibril type, except for telocollagen, whose mean adhesion was significantly lower than the others (Fig. S1†). This difference is not a height effect considering that atelo- and telocollagen have almost identical height ranges (Fig. 5a).

Using the adhesion fingerprints, the adhesion force experienced by the tip in the overlap and gap regions of the D-band was calculated (Fig. 5b) as shown in Fig. S2.† The average resultant adhesion force, *F*_D-band_ was positive for the gap and negative for the overlap region (Fig. 5b). In principle, this adhesion force measured by the AFM between the tip and the fibril is governed by three forces-the Van Der Waal's force (*F*_vdW_), the Capillary force (*F*_c_), and the Electrostatic force (*F*_e_) as represented by the equation below:

1 *F*_D-band_ = *F*_vdW_ + *F*_c_ + *F*_e_

Any of these forces, separately or together, can result in the adhesion contrast observed between the overlap and gap. The vander Waal's force, *F*_vdW_, is expected to be modulated by the molecular density, for which the electron density in Fig. 1 is a proxy. So, the higher the electron density, the greater *F*_vdW_ should be. If *F*_D-band_ is purely governed by *F*_vdW_, the adhesion force should be greater in the overlap compared to the gap region, as the overlap is denser than the gap (Fig. 1). However, this is the opposite of our experimental data (Fig. 5b). So, *F*_vdW_ does not appear to be the prime factor responsible for this contrast. Next, the capillary force, *F*_c_, is a function of humidity in the air^22^ and should play a role in modulating *F*_D-band_ as follows. Because of the tip geometry and the trough created in the gap region, water absorption is higher in the gap compared to the overlap,^22^ resulting in a greater capillary force in the former than the latter. Hence, *F*_c_ contributes to a higher adhesion in the gap than the overlap region, which is qualitatively consistent with our results (Fig. 5b). However, as the height difference between the gap and overlap regions increases, the capillary force in the gap region should increase. Yet, the mean adhesion values of the overlap and gap regions are not significantly different among the fibril types even though they cover a wide range of D-band amplitudes (Fig. 5a). Therefore it is unlikely that *F*_c_ alone drives the observed spatial modulation of the adhesion force.

Since neither *F*_vdW_ nor *F*_c_ can fully explain the results, the hypothesis we put forward is that the n-doped Silicon tip used in our experiment is acting like a negative charge. Since it has been previously shown using KPFM that the overlap (gap) region is negatively (positively) charged,^15^ following the hypothesis, the tip would be repelled (attracted) by the overlap (gap) region, hence yielding a negative (positive) adhesion. This is consistent with the results (Fig. 5b). So, the Electrostatic force between the tip and the sample, *F*_e_ can be one of the forces to be accounted for the adhesion pattern observed and is likely the main contributor to the observed sub-D-band force dips.

### 3.2 Molecular assignment of fibril type specific adhesion fingerprints

**Fig. 6:**
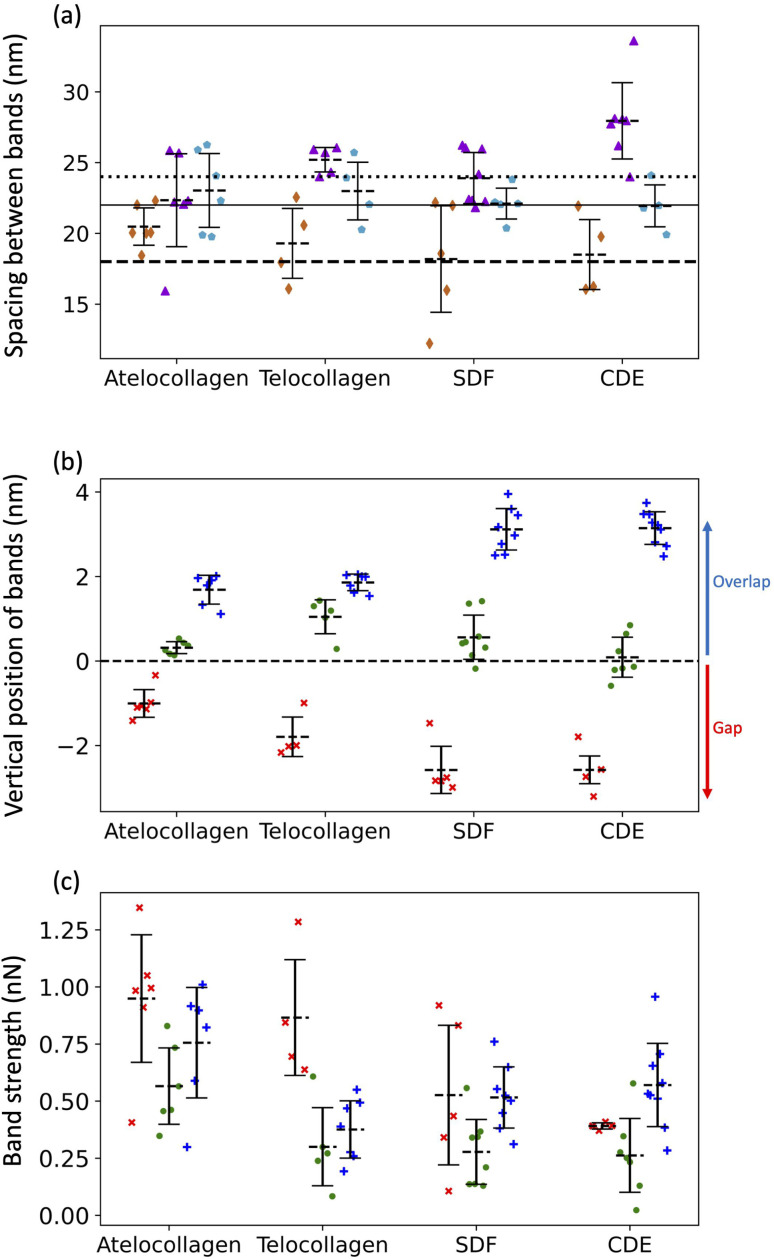
(a) Average spacing between X1 and X2 bands (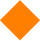), X2 and X3 bands (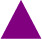) and X3 and X1 bands (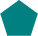) along with the spacing between X1 and X2 bands (dashed line), X2 and X3 bands (solid line) and X3 and X1 bands (dotted line) calculated using cryo-TEM data. (b) Height location of bands, X1 (×), X2 (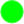) and X3 (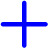). Negative height position (X1) indicates that the band is located in the gap region whereas positive height position (X2 and X3) indicates that the band is located in the overlap region. (c) Adhesion Strengths of X1 (×), X2 (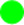), X3 (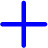) bands. See Fig. S2† for details.

Three adhesion force dips are consistently visible in the fingerprints of all four fibril types. We attribute these dips to the X1, X2, and X3 bands-the three main electron-dense regions observed by Quan and Sone using Cryo-EM (Fig. 1b and 4b). Other, less conspicuous bands were also visible, albeit less frequently in the fingerprints as compared to the unprocessed profiles as they are often lost due to averaging (Fig. S3†). The respective spacing between the three main bands for all fibril types approximately matches the ones observed by Cryo-EM (Fig. 1b and 6a). However, we also observe that each individual fibril has a unique set of spacing and that on average, each fibril type differs in spacing by a few nanometers. Taking advantage of the registration between the adhesion and height fingerprints, it was possible to locate the height position of each adhesion force dip along the D-band topography (Fig. 6b and S2†).

It has been well-documented that during fibril formation, a 3.8 nm lattice plane of collagen molecules orients radially, which causes the C-terminal telopeptide to be jutted outwards, causing it to be at the apex of the fibril's corrugated surface, and the N-terminal telopeptide to be buried underneath the fibril surface.^23,24^ Hence, the X3 band, which corresponds to the C-terminus of the collagen molecule, is located at the apex of the D-band repeat with the X2 band, corresponding to the N-terminus, located a few nanometers underneath (Fig. 6b), validating our band assignment.

From the results so far, these dips in the adhesion force, *F*_D-band_ can be attributed to two forces-the capillary force, *F*_c_, which is dependent on the topography, or the Electrostatic force, *F*_e_, which is dependent on surface charge. One possibility is that the visibility of these sub D-band features is a result of the modulation of *F*_c_ alone. While the most intense dips in adhesion match the electron-dense peaks, since *F*_c_ is dependent on topography, each dip in adhesion should be accompanied by a proportional variation in height.^22^ This, however, is not the case since there are many instances where the dips in the adhesion profile associated with the characteristic bands, X1, X2, and X3, are not accompanied by any peaks in the height profile (Fig. 4 and S3†). Moreover, *F*_c_ is also expected to be weaker in shallower dips of shorter, atelocollagen fibrils as compared to deeper dips of taller, CDE fibrils. But, since this was not observed in the results, variations in capillary force are unlikely to be the explanation for the detection of these sub-bands. Accounting for the other possibility, the X1, X2, and X3 bands marked in Fig. 4 also correlate to d, c, and a peaks, respectively, observed in the Uranyl Acetate stain profile (Fig. 1). These peaks represent regions with a high number of negative charges within the fibril. If they were net negatively charged, these regions would repel a negatively charged tip and thus decrease the electrostatic force, *F*_e_, resulting in a dip in adhesion force, *F*_D-band_. Hence, in addition to explaining the adhesion contrast observed between the overlap and gap, *F*_e_ could also be responsible for the visibility of these sub-bands.

### 3.3 Fibril types have intrinsic differences in the surface charge pattern

To compare the differences in adhesion measured among the fibril types at a higher resolution, adhesion strength for the three bands was measured, as shown in Fig. S2.† It was revealed that in addition to a unique set of spacing between the X1, X2, and X3 bands, each fibril type has a unique set of adhesion strengths for the three bands (Fig. 6c). Assuming that adhesion is purely governed by electrostatic force and interpreting adhesion band strength as a proxy for the net negative surface charge, we observe that the X2 band (N-terminus) is on average less negatively charged than the X3 band (C-terminus) for all fibril types (Fig. 6c) and that there is significant intra-fibril variation (Fig. 4b gray shading and S3†). Comparing across fibril types, the strengths of the X2 and X3 bands are basically identical for SDF and CDE fibrils (Fig. 6c). This is expected considering that in both cases, the N- and C-telopeptides are engaged in crosslinks with adjacent collagen molecules.^6^ The telocollagen fibrils have an X2 band identical to the SDF and CDE fibril but a lower X3 band (Fig. 6c). This is consistent with the idea that the N-terminal telopeptide is buried under the fibril surface even for *in vitro* assembled fibrils whereas the C-terminal telopeptide is exposed at the surface and interacting with adjacent molecules in a non-native way. In the atelocollagen case, where both telopeptides are missing, the X2 and X3 bands are stronger than for the three other types indicating a unique conformation of the collagen molecules at the fibril surface in two regions that host interacting sequences with several key collagen partners such as keratan sulfate, metalloproteinases, fibronectin and integrins.^24^

The strength of the X1 band is also worth analyzing because it is the *in vivo* interaction site for another proteoglycan, decorin.^25^ For all fibril types, the X1 is a negative dip in adhesion force superimposed on one side of the positive adhesion force peak characteristic of the gap region (Fig. 4b). Because we use the maximum adhesion force in the gap region as our reference (Fig. S2†), we likely overestimate the strength of the X1 for all fibril types, including CDE. Nonetheless, all fibrils are analyzed in the same way, so the decrease in strength of the X1 band for the SDF and CDE fibrils compared to the *in vitro* assembled ones may be due to the presence of decorin molecules at the surface of the SDF and CDE fibrils. Decorin is composed of a positively charged core protein domain that covers the X1 band and one negatively charged chondroitin sulfate chain.^26^ The mechanical extraction of the fibrils from the SDF and CDE tendons is expected to remove some if not all chondroitin sulfate chains, leaving only the decorin core covering the X1 band. We propose that the CDE fibrils have had their chondroitin sulfate chains consistently removed, thus producing an X1 band of low but very well-defined strength (Fig. 6c). SDF fibrils in comparison have a much broader range of X1 band strength that is likely a result of the higher cohesiveness between fibrils in this tissue,^27^ thus requiring a more forceful extraction than in the CDE case. Finally, the increase in strength of the X1 band for the *in vitro* assembled fibrils compared to the SDF and CDE (Fig. 6c) is likely due to the absence of the decorin core protein.

There are different reasons which can be responsible for a discrepancy between our results and the ones obtained by Quan and Sone using Cryo-EM.^7^ The electron density used as reference (Fig. 1) was measured on a single fibril extracted from a rat tail tendon which is similar to a bovine CDE tendon. As explained above, there are many intrinsic differences between fibrils of different types and hence, results obtained from each of them cannot be expected to be entirely similar. Moreover, cryo-EM reveals details in the bulk of a material unlike the AFM, which displays surface properties. So, a discrepancy between the results obtained by the two methods can be an indication of how the charge distribution on the surface of the fibril is different from its interior.

### 3.4 Fibril orientation and out-of-plane molecular tilt

**Fig. 7:**
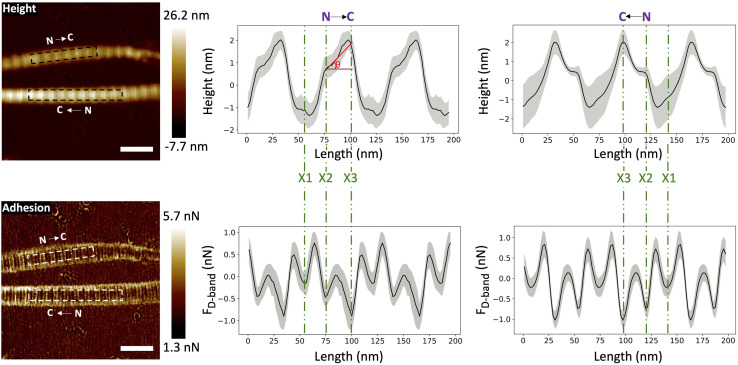
Height and adhesion images of two fibrils with opposite polarity. The arrows on each image indicate the orientation from the N-terminus to the C-terminus of the collagen molecules within the fibrils. Height and adhesion fingerprints extracted from left to right in the broken line boxes are presented with the assignment of the X1, X2 (N-terminus) and X3 (C-terminus) bands. The angle of out-of-plane molecular tilt between the N- and C-termini, (θ), is defined as shown. (Scale bar = 200 nm).

Within a collagen fibril, all the molecules are oriented in the same direction, making the structure polar.^28^ Assessing this polarity on a fibril-by-fibril basis is experimentally challenging. Negative staining electron microscopy,^2^ Piezoelectric Force Microscopy^29^ and second harmonic generation microscopy^30,31^ are the three main techniques capable of identifying polarity, absolutely in the first case and comparatively in the other two cases. Leveraging our assignment of the X1, X2 and X3 bands in the height and adhesion fingerprints, it is possible to define the polarity of a collagen fibril in absolute term (Fig. 7) without staining.

**Fig. 8:**
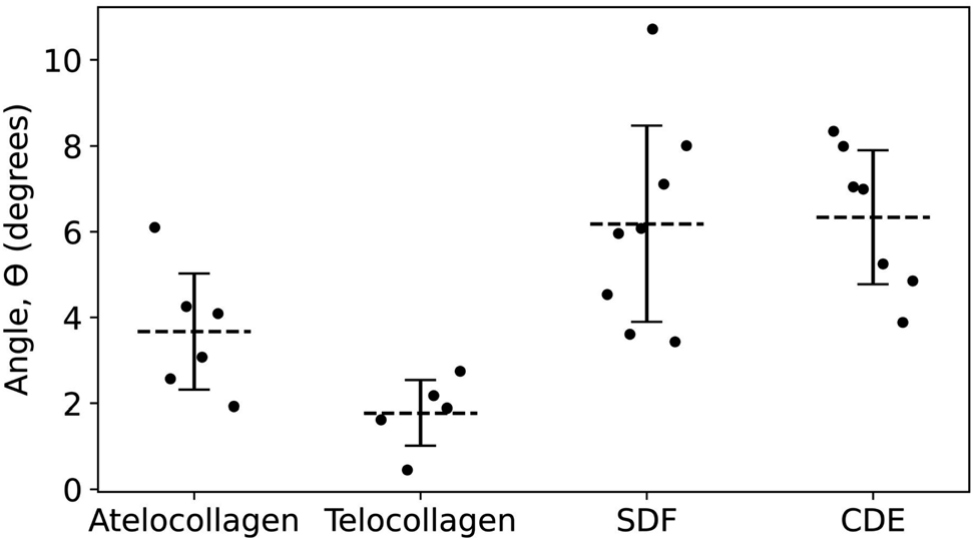
Angle of out-of-plane molecular tilt between the N- and C-termini (Fig. 7) for the four different fibril types.

In principle, this approach could be combined with imaging of tissue cryo-sections to characterize the polarity of collagen fibril bundles in skin or tendons.^32^ Furthermore, with the polarity identified, it is possible to measure the out-of-plane molecular tilt, predicted by Orgel and coworkers,^33^ using the positions of the X2 and X3 bands in the height fingerprint as shown in Fig. 7. Telocollagen fibrils have the lowest average out-of-plane molecular tilt, approximately 2°, followed by atelocollagen fibrils with similar height but an average tilt of 4° (Fig. 8). This factor of two in molecular tilt further demonstrates the subtle differences in fibril structure induced by the deletion of the telopeptide as already observed with the adhesion strength of the three bands (Fig. 6c). The two types of *ex vivo* fibrils have the largest average out-of-plane molecular tilt, 6° (Fig. 8) which is likely set by the presence of enzymatic cross-links at the C-terminus.^24^ In all four cases, the measured angle is affected by changes in the shape of the fibrils as they dry on the glass substrate.^34^

## 4 Conclusions

In this study, we investigated the modulation of adhesion force measured between an AFM tip and collagen fibrils and discussed how various interactions may affect this force to demonstrate that adhesion force microscopy is likely sensitive to the presence of charges implanted at the surface of a material even in the absence of an applied voltage. Following this idea, different experiments can be performed to further understand the contribution of electrostatic interactions on adhesion force measured by the AFM, which would yield a novel way to accurately map the charge distribution on a surface, with a resolution of less than 20 nm. Taking advantage of the periodic nature of the fibril surface, we also reveal subtle differences in fibril architecture and possibly surface charge distribution for *in vitro* and *in vivo* assembled fibrils. In principle, our approach is capable of characterizing changes in surface charge distribution induced by bound proteins such as decorin or non-enzymatic cross-links like advanced glycation end products that accumulate as collagenous tissues age. It also breaks new ground in the measurement of charges distribution on other solid surfaces of materials such as polymers or polyelectrolytes.

## Author contributions

Vinayak Mull: conceptualization, data curation, formal analysis, investigation, methodology, software, validation, visualization, writing – original draft. Laurent Kreplak: conceptualization, funding acquisition, methodology, project administration, resources, supervision, writing – review editing.

## Conflicts of interest

There are no conflicts to declare.

## Supplementary Material
